# Association of Homocysteine with Aysmptomatic Intracranial and Extracranial Arterial Stenosis in Hypertension Patients

**DOI:** 10.1038/s41598-017-19125-9

**Published:** 2018-01-12

**Authors:** Yan Wang, Jin Zhang, Yuesheng Qian, Xiaofeng Tang, Huawei Ling, Kemin Chen, Yan Li, Pingjin Gao, Dingliang Zhu

**Affiliations:** 1 Research Center for Hypertension Management and Prevention in Community, Shanghai Key Laboratory of Hypertension, Shanghai Institute of Hypertension, State Key Laboratory of Medical Genomics, Ruijin Hospital, Shanghai Jiaotong University School of Medicine, Shanghai, China; 2 Department of Radiology, Ruijin Hospital, Shanghai Jiaotong University School of Medicine, Shanghai, China

## Abstract

Elevated plasma homocysteine (Hcy) is suggested as an independent risk factor for stroke. We aimed to investigate the association of Hcy concentration with intracranial atherosclerosis (ICAS) and extracranial AS (ECAS) in hypertensive patients without stroke in Chinese population and to explore modified effect of methylenetetrahydrofolate reductase (MTHFR) C677T on their relationship. The stenosis of intracranial and extracranial arteries were evaluated in a total of 929 subjects through computerized tomographic angiography (CTA) from aortic arch to the skull base. Hcy concentration showed significantly association with both ICAS (OR: 1.105; 95% CI: 1.057–1.155) and ECAS (OR: 1.096; 95% CI: 1.047–1.146) for 1 µmol/L increment in Hcy. Meanwhile, hyperhomocysteinemia (≥15 µmol/L) was also displayed association with ICAS (OR: 1.587; 95% CI: 1.029–2.446) and ECAS (OR: 2.164; 95% CI: 1.392–3.364) after fully adjustment. Furthermore, in the subgroup analysis, such association remained significant only in the subjects that were younger, with normal renal function and with MTHFR 677 C allele. Our study showed the significant association of Hcy with ECAS and ICAS in asymptomatic hypertension patients. Hcy played a universal effect on the cervico-cerebral atherosclerosis. Such association was modified by the MTHFR C677T genotype.

## Introduction

Stroke ranks the leading cause of permant disability in China, with the disability-adjusted life-years rates nearly twice as high as ischemic heart disease^1^. Atherosclerosis is a well-known risk factor for stroke in both western and eastern population. But the distribution of atherosclerosis seems different among vulnerable individuals, whereas intracranial arterial stenosis (ICAS) is more common in Asians, blacks and Hispanics, while extracranial arterial stenosis (ECAS) is more popular among whites^2,3^.

Homocysteine (Hcy), a homologue of the amino acid cysteine, is a sulfurcontaining amino acid, which can be recycled to methionine or trans-sulfurated to cystathionine required for pyridoxial-5phosphate^4^. Raised concentration of Hcy has been suggested as an independent risk factor for stroke, especially in the Chinese hypertension patients^5^. The concentration of Hcy is known to be influenced by a common variant of methylenetetrahydrofolate reductase (MTHFR) C677T^6^, in which T/T genotype leads to a reduction in enzyme activity, resulting in decreased folate level and increased Hcy. It has been reported that the effect of Hcy on stroke was siginificantly modified by the MTHFR C677T genotype^7^. However, there is little study focusing on the joint effect of Hcy and MTHFR C677T with ICAS or ECAS. Hypertension has been associated with both ischemic and hemorrhagic forms of stroke, as well as with ICAS and ECAS^8,9^. We hereby aim to investigate the association of Hcy concentration with ICAS and ECAS in hypertensive patients without stroke in Chinese population; furthermore, we sought to examine the effect of MTHFR C677T on their relationship.

## Method

### Study design

This study was an ongoing cross-section and prospective study in China, which was to detect the relationship between intra- and extracranial asymptomatic artery stenosis and stroke outcome in stroke-free hypertension patients through computerized tomographic angiography (CTA). All the participants were recruited from hypertension outpatients who were identified in Xinzhuang Community hospital between May 2012 and December 2015, and then referred to Ruijin Hospital for the baseline and follow-up studies. Patients with hypertension were enrolled if they had ≥2 cardiovascular risk factors defined according to the Chinese hypertension guidelines^10^ and were willing to undergo an examination of brain using CTA and to participate in long-term follow-up of their health. The subjects with blood pressure (BP) ≥ 140/90 mmHg, or taking antihypertensive medication were considered as hypertension patients. The major exclusion criteria included history of stroke, transient ischemic attack, atrial fibrillation and iodine allergy.

All methods were performed in accordance with the relevant guidelines and regulations.

### Ethics Statement

The study protocol was approved by the ethics committee of Ruijin Hospital and written informed consent was obtained from all participants.

### Demographic and clinical measurements

BPs were measured in the sitting position using a verified electronic sphygmomanometer (OMRON, HEM-907) by a trained physician or nurse, after a 5-mins rest. The average of three consecutive BP readings with one-minute interval in the first visit of each participant was used for the current analysis. Body weight and height were recorded with participants wearing light indoor clothing and no shoes. The health-related behavior and medical history were collected by interview. Current smokers were defined as those who had smoked cigarettes on one or more days in the past 30 days. All the biochemical measurements were performed in the Central Laboratory of Ruijin Hospital (Shanghai, China) using the standard protocols, including serum concentrations of fasting plasma glucose (FPG), serum lipid and creatinine (Scr). Serum homocysteine (Hcy) level was measured by enzymatic cycling method (test kit provided by Axis-Shield Diagnostics Ltd) with Beckman Coulter AU5800 biochemical analyzer (USA, with the repeatability CV as 1.8%).

### CTA protocol

CTA acquisitions were performed as previous described^11^ (GE FX/I, General Electric, Fairfield, CT) according to an established protocol. Five-millimeter helical cuts were made starting from the aortic arch to the skull base. Images were reformatted with 1-mm slice thickness. Two experienced radiologist blinded to clinical information independently reviewed all the CTA images at a workstation with the software of AW4.4 vessel analysis. The percentage of stenosis was calculated as the ratio of the smallest diameter of the lesion segment divided by the diameter of a nearby normal segment. The intracranial arteries included intracranial segment of internal carotid artery and vertebral artery, basilar artery, anterior cerebral artery, middle cerebral artery and posterior cerebral artery. The extracranial arteries included extracranial segment of internal carotid artery and vertebral artery, external carotid artery, common carotid artery and subclavian artery. The two radiologists had good agreement in the designation of stenosis (κ = 0.93, P < 0.001). All disagreements were reviewed and adjudicated by a senior radiologist to reach a consensus.

### Genotyping

DNA was extracted from the blood samples using a FU-JIFILM QuickGene-610L system. The MTHFR C677T genotyping work was performed using a custom-by-design 48-Plex SNPscan™ Kit (Cat#: G0104K, Genesky, Shanghai, China), which was developed according to patented SNP genotyping technology based on double ligation and multiplex fluorescence PCR. Each 96-well plate included 1 non-template control. For quality control, repeated analyses were performed for 4% of randomly selected samples with high DNA quality.

### Statistic Analysis

SPSS software (version 13.0; SPSS Inc., Chicago, Illinois, USA) was used to manage the database and analyze. The comparisons of patients with or without ICAS were performed using a Pearson Chi-square test for categorical variables and Student t test for continuous variables. Stepwise logistic regression in forward conditional method was used to detect the association of homocysteine (1 µmol/L) and hyperhomocysteinemia (>15 µmol/L) with ICAS and ECAS in three models. The basically adjusted association was adjusted for sex and age, and fully adjusted association was adjusted for body mass index (BMI), current smoking, diabetes, LDL, SBP, Scr, anti-hypertensive treatment, and statin used in addition. All *P* values were 2-tailed, and a *P* value of <0.05 was considered statistically significant.

## Results

### Characteristics of the Study Participants

A total of 929 participants were enrolled in the current study, among which 432 were absent of ICAS or ECAS, 143 had ECAS only, 198 had ICAS only, and 156 had concurrent extraintracranial artery stenosis (Table 1). The patients with ICAS were older, with higher level of SBP, FSG, LDL, Scr, Hcy, and higher frequency of male, diabetes and anti-hypertensive treatment. The patients with ECAS were older, with lower DBP, and with higher level of LDL, Scr and Hcy. The subjects with either ECAS or ICAS showed higher frequency of male, smoking, diabetes, and anti-hypertensive treatment, and had higher SBP, FPG, LDL, Scr and Hcy, but lower DBP.

**Table 1:** Clinical characteristics of hypertensive patients according to location of arterial stenosis.

	ICAS/ ECAS absent	ICAS present	ECAS present	COMB present	All stenosis
N	432	198	143	156	497
Age (years)	62.9 ± 5.8	65.4 ± 5.8^†^	65.4 ± 5.7^†^	67.2 ± 5^†^	66.0 ± 5.6^†^
Male (N,%)	158 (36.6)	105 (52.5)^†^	70 (49.0)*	95 (60.9)^†^	270 (54.1)^†^
Smoking (N,%)	54 (12.5)	33 (16.5)	23 (16.1)	30 (19.2)*	86 (17.2)*
Body mass index (kg/m^2^)	25.0 ± 3.1	25.4 ± 3.0	25.0 ± 3.1	25.4 ± 2.9	25.3 ± 3.0
Office SBP (mmHg)	134.4 ± 15.6	139.6 ± 16.1^†^	136.4 ± 16.0	139.6 ± 17.4^†^	138.6 ± 16.5*
Office DBP (mmHg)	73.2 ± 10.0	73.4 ± 10.2	70.8 ± 10.2*	70.9 ± 9.6*	71.8 ± 10.1*
Plasma glucose (mmol/L)	5.1 ± 1.1	5.4 ± 1.6*	5.2 ± 1.2	5.5 ± 1.5*	5.4 ± 1.5*
Total cholesterol (mmol/L)	4.8 ± 0.8	4.8 ± 0.9	5.0 ± 0.9	4.9 ± 0.9	4.9 ± 0.9
Low-density lipoprotein (mmol/L)	2.8 ± 0.8	3.0 ± 0.8*	3.0 ± 0.7*	3.0 ± 0.8*	3.0 ± 0.8*
Serum creatinine (µmol/L)	63.1 ± 16.6	67.0 ± 17.1*	67.4 ± 17.2*	68.8 ± 17.8^†^	67.7 ± 17.4
Serum homocysteine (µmol/L)	11.9 (10.2–14.3)	13.5 (11.1–16.0)^†^	13.3 (10.7–16.3)^†^	13.6 (11.5–16.3)^†^	13.5 (11.2–16.2)^†^
Diabetes mellitus (N,%)	77 (17.8)	54 (27.0)*	26 (18.2)	43 (27.6)*	123 (24.6)*
Anti-hypertensive treatment (N,%)	365 (84.7)	183 (91.5)*	128 (89.5)	143 (91.7)*	494 (91.0)*
Statin use (N,%)	29 (6.7)	12 (6.0)	11 (7.7)	22 (14.1)*	45 (9.0)
MTHFR C677T					
CC	74 (17.9)	27 (14.7)	18 (13.1)	21 (14.3)	66 (14.1)
CT	186 (44.9)	96 (52.2)	65 (47.4)	72 (49.0)	233 (49.8)
TT	154 (37.2)	61 (47.2)	54 (39.4)	54 (36.7)	169 (36.1)

Data are expressed as mean ± SD, or percentage (%).ECAS, extracranial arterial stenosis; ICAS, intracranial arterial stenosis; COMB, combined extra- and intracranial arterial stenosis. SBP, systolic blood pressure; DBP, diastolic blood pressure. P indicates the comparison among four groups. *p < 0.05 and ^†^p < 0.01 in comparison with ICAS/ ECAS absent group.

### Hcy and ICAS/ECAS

In unadjusted continuous analysis, Hcy concentration was significantly associated with both ICAS (OR: 1.105; 95% CI: 1.057–1,155) and ECAS (OR: 1.096; 95% CI: 1.047–1.146) for 1 µmol increment in Hcy. Estimates remained statistically significant after fully adjustment, with the risk of ICAS and ECAS increased by 5.7% and 8.0% for each 1 µmol/L increment of Hcy concentration (Table 2).

**Table 2:** Continuous and Binary Analysis on the Association of serum homocysteine and Stenosis in Intracranial and Extracranial Ateries.

	Model I		Model II		Model III	
	OR(95%CI)	P value	OR(95%CI)	P value	OR(95%CI)	P value
**Isolated ICAS**						
Homocysteine (+1 µmol)	1.105(1.057–1.155)	<***0***.***001***	1.077 (1.028–1.138)	***0***.***002***	1.057 (1.004–1.113)	***0***.***034***
Homocysteine >15 µmol	2.150(1.466–3.155)	<***0***.***001***	1.554 (1.019–2.371)	***0***.***041***	1.587 (1.029–2.446)	***0***.***037***
**Isolated ECAS**						
Homocysteine (+1 µmol)	1.096(1.047–1.146)	<***0***.***001***	1.072 (1.022–1.123)	***0***.***004***	1.080(1.029–1.133)	***0***.***002***
Homocysteine >15 µmol	2.439(1.603–3.711)	<***0***.***001***	2.040 (1.321–3.150)	***0***.***001***	2.164 (1.392–3.364)	***0***.***001***
**All stenosis**						
Homocysteine (+1 µmol)	1.114 (1.074–1.155)	<***0***.***001***	1.057 (1.015–1.110)	***0***.***007***	1.056 (1.014–1.100)	***0***.***008***
Homocysteine >15 µmol	2.308(1.701–3.132)	<***0***.***001***	1.585 (1.127–2.229)	***0***.***008***	1.591 (1.125–2.251)	***0***.***009***

OR, odds ratio. Model I crude association. Model II adjusted for age and sex. Model III adjusted for age, sex, BMI, current smoking, diabetes, Low-density lipoprotein, systolic blood pressure, serum creatinine, anti-hypertensive treatment, and statin use.

In unadjusted binary analysis, the hyperhomocysteinemia were both significantly associated with ICAS and ECAS (P < 0.001). After adjustments for all of the confounding factors, the strength of the association of hyperhomocysteinemia with ICAS was attenuated but still significant (OR = 1.587, 95% CI: 1.029–2.446), It was also the case for hyperhomocysteinemia and ECAS, with the corresponding OR = 2.164 (95% CI, 1.392–3.364) after fully adjustment.

**Figure 1:**
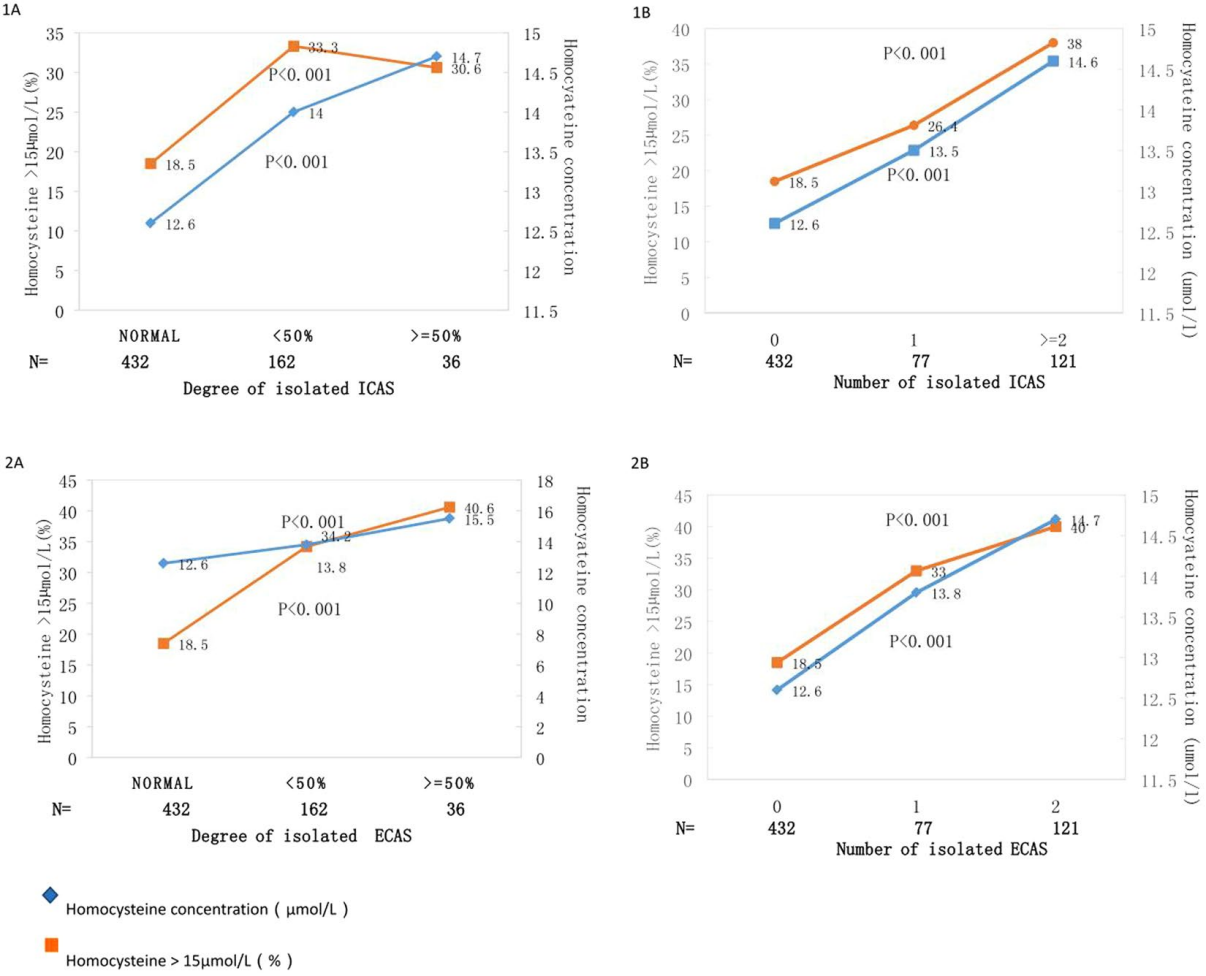
Homocysteine concentration (µmol/L) and prevalence of Homocysteine >15 µmol/L (%) according to the degree and number of isolated intracranial arterial stenosis (1A & 1B) and the degree and number of isolated extracranial arterial stenosis (2A & 2B). P values are for the comparison between the groups.

In concern with the severity and distribution of arterial stenosis, the patients with moderate to severe stenosis had higher Hcy concentration and higher prevalence of hyperhomocysteinemia (P < 0.001). Additionally, the patients with more vessels injured also demonstrated higher Hcy concentration and higher rate of hyperhomocysteinemia (P < 0.001) (Fig. 1).

### MTHFR C677T and ICAS/ECAS, Hcy

There was a significant difference in Hcy among MTHFR C677T genotypes: median (IQR) plasma Hcy for the CC genotype was 12.5 (10.4–14.8); the CT genotype, 12.7 (10.7–15.1); the TT genotype, 13.4 (11.0–18.4) µmol(P < 0.001). However, this polymorphism was not associated with either isolated ICAS (P = 0.250) or ECAS (P = 0.436). Furthermore, it was not directly related to the severity of stenosis in intracranial (P = 0.426) or extracranial arteries (P = 0.257).

### Sensitivity Test

We also tested the association in subgroups according to various baseline characteristics (Table 3). Since there was no significant difference of Hcy among the patients with ICAS, ECAS or concurrent extraintracranial artery stenosis, we combined the patients of these three groups in to one and compared them with the subjects absent of stenosis. The relationship between Hcy and arterial stenosis was dependent on age. The Hcy showed a significant association with arterial stenosis in those younger than 65 years both in binary and continuous regressions. Additionally, in the subjects with good renal function (eGFR >  = 90 mL/min/1.73 m^2^), the association of Hcy and arterial stenosis remained significant, while it was not the case for those with impaired renal function. We also detected a significant influence of MTHFR C677T on Hcy effect. Among the participants with CC/CT genotype but not TT genotype, the Hcy associated with arterial stenosis significantly.

**Table 3:** Associations of homocysteine with arterial stenosis in subgroup analysis.

	Homocysteine (+1 µmol)		Homocysteine > 15 µmol	
	OR (95% CI)	P	OR (95% CI)	P
Men (N = 426)	1.049 (0.995–1.106)	0.077	1.509 (0.969–2.351)	0.069
Women (N = 503)	1.066 (0.992–1.146)	0.081	1.505 (0.793–2.855)	0.211
Age < 65 y (N = 450)	***1***.***093*** (***1***.***021–1***.***169***)	***0***.***010***	***1***.***911*** (***1***.***091–3***.***346***)	***0***.***023***
Age > = 65 y (N = 479)	1.027 (0.973–1.085)	0.333	1.298 (0.806–2.090)	0.284
eGFR > = 90 mL/min/1.73 m^2^ (N = 718)	***1***.***059*** (***1***.***004–1***.***116***)	***0***.***035***	***1***.***709*** (***1***.***089–2***.***683***)	***0***.***020***
eGFR < 90 mL/min/1.73 m^2^ (N = 211)	1.044 (0.971–1.123)	0.247	1.239 (0.650–2.362)	0.515
MTHFR 677 CC/CT (N = 742)	***1***.***096*** (***1***.***033–1***.***164***)	***0***.***002***	***1***.***767*** (***1***.***136–2***.***746***)	***0***.***011***
MTHFR 677 TT (N = 140)	1.071 (0.99–1.159)	0.089	1.472 (0.626–3.460)	0.375

All the P value was adjusted for age, sex, BMI, current smoking, diabetes, Low-density lipoprotein, systolic blood pressure, serum creatinine, anti-hypertensive treatment, and statin use.

## Discussion

In the present study, we found the association of Hcy with both ICAS and ECAS in an asymptomatic Chinese hypertension population, who underwent CTA examination in intra- and extra- cranial arteries simultaneously. Subjects with higher Hcy showed more sever stenosis and had more arterial lesions in both intra- and extracranial arteries. Furthermore, in the subgroup analysis, such association remained significant only in the subjects that were younger, with normal renal function and with MTHFR 677 C allele.

No previous study has investigated the effect of plasma Hcy and MTHFR C677T on ICAS and ECAS evaluated by CTA in asymptomic hypertension patients. Kim *et al*. had analyzed the relationship of Hcy, MTHFR C677T and ICAS & ECAS in ischemic stroke patients by magnetic resonance angiography (MRA), but no significant result was found^12^. Since MRA was only able to detect the severe stenosis ( >50%), it might underestimate the prevalence of ICAS and ECAS. CTA had been proved to provide better delineation of the anatomy of intra- and extracranial arteries with DSA than TCD or MRA^13–15^, which guaranteed the diagnostic accuracy and sensitivity of the luminal stenosis.

Since the first report about the association of plasma Hcy concentration and ECAS^16^, amount of studies were carried out to test the relationship of carotid plaques, intima-media thickness and Hcy concentration, but the results was not consistent in all studies. In a population-based multi-ethnic cohort, Sara *et al*. found the elevated Hcy was independently associated with plaque morphology and increased plaque area^17^. In another community-based study with 5359 Chinese participants, Hcy was found as an independent indicator of asymptomatic ECAS, especially in patients with diabetes. However, the results from large cohort of Framingham Offspring and Nothern Manhattan did not support this finding^18,19^. There were several possible reasons for the inconsistent finding, including ethic differences, the accompanied diseases, methods used to assess the degree of ECAS and the folic acid supplementation. The level of Hcy was higher in hypertension patients^20–22^, and the risk of cardiovascular disease was stronger in the subjects with hypertension combined with hyperhomocysteinemia^23^. But there was few studies focusing on the effect of Hcy on ECAS in hypertension patients. The study of Catena *et al*. was the only one which had explored the relationship of Hcy and ECAS in hypertension patients with relative large sample size, and got the similar result with us, which indicated a role of Hcy in the development and progression of subclinical carotid atherosclerosis in hypertension^24^. The Hcy lowering treatment was proved to be more effective in stroke primary prevention, especially in the regions with low folate^25^. We performed the study in asymptomatic hypertension patients without mandatory folic acid fortification in Chinese population, which might reveal the influence of Hcy on ECAS to the great extent. Additionally, most of the previous studies focused on isolated ECAS or ICAS, which might included some subjects with atherosclerosis in other part of carotid system in controls and then weaken the ability to detect the association.

Comparing to the research on ECAS, there were few studies focusing on the relationship of ICAS and Hcy, especially in asymptomatic subjects. Park *et al*. had retrospectively analyzed brain MRA in 682 non-stroke individuals, and found that the elevated plasma Hcy levels were associated with small vessel disease (SVD) but not cervico-cerebral atherosclerosis, regardless of the location of the atherosclerosis^26^. While, a study involved 825 noncardioembolic ischemic stroke patients found that hyperhomocysteinemia was associated with both SVD and cervico-cerebral atherosclerosis^27^. The few participants and the low prevalence of ICAS might impair the ability to find the underlying relationship. In the present study, we enrolled 929 patients with the prevalence of isolated ICAS of 21.3%, which ensured us to explore the association of Hcy and ICAS.

Although atherosclerosis can affect all arteries in the body, the distribution and severity of atherogenesis varied among different populations, whereas ICAS is more common in Asians, blacks and Hispanics^2^, while ECAS is more popular among whites^28^. Several previous studies had showed that ICAS and ECAS might involved different risk factors^29,30^. In our previous study, we also found a significant and independent association of Lp-PLA_2_ mass with ICAS, but not with ECAS^11^. However, the effect of Hcy on ICAS and ECAS seemed similar in our results. Although the ICAS was more popular in Chinese population and Hcy was considered as a risk factor of ischemic stroke, Hcy seemed performed a universal influence on the atherosclerosis in ICA and ECA.

The modifying effect of MTHFR C677T had been reported by several studies. Zhao *et al*. found that baseline Hcy was associated with an increased risk of first stroke among participants with the CC/CT genotype, but not among participants with the TT genotype^7^. In the SHEEP study, the association between Hcy and myocardial infarction was observed only in MTHFR 677 C carrier, but not those with T homozygote^31^. Such results were in accordance with our finding, and indicated interplay of MTHFR genotype and Hcy on the formation of cervico-cerebral atherosclerosis.

Several limitations of the present study should be mentioned here. Firstly, as the present study is cross-sectional designed, we could not infer any causal relationship between Hcy and ICAS/ECAS. Secondly, we did not systematically measure folate and vitamin B6 that could affect Hcy, which might bias the association. However, we performed this study in the Chinese population without folic acid fortification and all the patients had denied the intake of folic acid in the questionnaire, so that the low level of folate and vitamin B6 would have less impact on the Hcy. Thirdly, the distribution of arterial stenosis in the whites is different from in Asians, so that our finding might not be directly generalized to other populations.

In summary, we reported a significant association of Hcy with ECAS and ICAS in asymptomatic hypertension patients. Hcy played a universal effect on the cervico-cerebral atherosclerosis. Such association was modified by the MTHFR C677T genotype. Moreover, the mechanism and long-term effect of Hcy on the asymptomatic ICAS/ECAS still needed further studies.
